# A Novel Remote-Controlled Vascular Interventional Robotic System Based on Hollow Ultrasonic Motor

**DOI:** 10.3390/mi13030410

**Published:** 2022-03-04

**Authors:** Qing Lu, Zhijun Sun, Jialiang Zhang, Jiacheng Zhang, Juju Zheng, Feng Qian

**Affiliations:** State Key Lab of Mechanics and Control of Mechanical Structures, School of Aeronautics, Nanjing University of Aeronautics and Astronautics, Nanjing 210016, China; lq331572639@163.com (Q.L.); zhangjialiang2121@163.com (J.Z.); qq806627770@163.com (J.Z.); nuaazhengjuju@163.com (J.Z.); nuaa_qf@nuaa.edu.cn (F.Q.)

**Keywords:** master-slave robotic system, vascular interventional surgery, hollow ultrasonic motor, magnetic resonance imaging

## Abstract

Cardiovascular diseases (CVDs) are the deadliest diseases worldwide. Master-slave robotic systems have been widely used in vascular interventional surgery with the benefit of high safety, efficient operation, and procedural facilitation. This paper introduces a remote-controlled vascular interventional robot (RVIR) that aims to enable surgeons to perform complex vascular interventions reliably and accurately under a magnetic resonance imaging (MRI) environment. The slave robot includes a guidewire manipulator (GM) and catheter manipulator (CM) that are mainly composed of a hollow driving mechanism and a linear motion platform. The hollow driving mechanism is based on a traveling wave-type hollow ultrasonic motor (HUM) which has high positional precision, fast response, and magnetic interference resistance and realizes the cooperation of the guidewire and catheter by omitting the redundant transmission mechanism and maintaining good coaxiality. The HUM stator, the core part of the RVIR, is optimized by an adaptive genetic algorithm for better quality and greater amplitude of traveling waves, which are beneficial to the drive efficiency and precision. The robot system features great cooperating performance, small hysteresis, and high kinematic accuracy and has been experimentally verified for its capability to precisely manipulate the guidewire and catheter.

## 1. Introduction

CVDs that lead to heart attacks and strokes have been increasing in recent years. According to a survey of the World Health Organization, more than 17 million people are killed by CVDs every year [1,2]. Minimally invasive surgery has become the main treatment method for CVDs due to the advantages of smaller incisions, shorter recovery time, and less blood loss [3,4]. Traditional vascular interventional surgery has high risk [5,6], such as unavoidable physiological tremors during operation, and needs well-skilled surgeons who require a long period of professional training and rich clinical experience. Therefore, numerous studies have been conducted on the application and development of RVIRs which can replace manual intervention [6,7,8,9,10,11,12]. However, RVIRs need to use real-time imaging which can be obtained by an X-ray or MRI to direct the guidewire and catheter.

Future directions are towards MRI-compatible devices because MRI can offer excellent image contrast for cardiovascular soft tissues [6,13,14,15,16,17] and avoid exposing patients and clinicians to harmful radiation as generated by X-rays [12,18,19,20]; these benefits offer the potential to improve the therapy strategy and enhance procedure efficiency and outcome [21,22,23,24]. However, compatible MRI vascular interventional master-slave robotic systems are only in their infancy. Lee et al. [12] developed a hydraulically driven vascular interventional robot system using distilled water as the transmission medium. The linear and twist motion of the catheter was realized by a piston and gear-rack mechanism. Abdelaziz et al. [6] developed a pneumatic driven vascular interventional delivery mechanism which adopts a pneumatic linear stepper motor and pneumatic rotary stepper motor to realize linear motion and rotary motion of the guidewire and catheter, respectively. Compared with a pneumatic drive, the incompressibility of liquid in the hydraulic drive is significantly better than that of gas, thus providing a more stable transmission and faster response. However, because the hydraulic driven device adopts a gear-rack mechanism to transfer the motion form, it brings the motion lag problem caused by gear clearance. In order to reduce the gear backlash, it is necessary to increase the pre-pressure exerted by hydraulic transmission on the gear, but this may lead to severe friction on the gear and pivot, which reduces the operating accuracy. In addition, hydraulic and pneumatic drives need electromagnetic motors to provide the remote power supply, in order to avoid the adverse effects of an electromagnetic motor on MRI imaging. The long-distance (>10 m) power transmission may cause large pressure loss and driving lag. This hysteresis phenomenon will significantly affect the performance of the robot system and would make it difficult to meet the high precision requirements of vascular interventional surgery. Tavallaei et al. [25] proposed an MRI-compatible remote catheter navigation system with 3 degrees of freedom which can realize the axial, rotation, and plunger actuation motion of the catheter by an ultrasonic motor, differential gear mechanism, rotating gantry, and winch-spring mechanism. This robotic system has a complex structure and contains a large number of intermediate transmission mechanisms which makes it difficult to achieve high-precision motion control. Liu et al. [26] presented the three-dimensional kinematic modeling of a steerable robotic ablation catheter system. The catheter, embedded with a set of current-carrying micro-coils, can realize the deflection motion by the magnetic force generated by the magnetic field of the MRI scanner. However, this driving method can only be used to drive the catheter to achieve a single bending deformation and cannot realize the axial and rotary motion, so it is difficult to be applied in vascular interventional surgery.

In addition, for complex vascular interventional surgery, such as cerebrovascular and vascular tumor surgery, the application of an active catheter that can rotate its guiding tip is greatly limited due to its size and safety problems [27,28]. Therefore, this surgery needs a surgeon to operate the passive catheter and guidewire simultaneously. In the vicinity of narrow vessel branches, the guidewire and passive catheter need to be pushed, pulled, and rotated to get them into the correct path; this operation process requires the cooperation of the guidewire and passive catheter. However, the full cooperation of the guidewire and catheter is rarely able to be realized due to the design limitations at present. For example, the long transmission chains of traditional robotic systems make catheters and guidewire difficult to meet the coaxial requirement, which may affect the cooperation accuracy [27] and cause additional wear. The Amigo, Corpath200, and Sensei Robotic Catheter Systems can only control the guidewire or catheter individually [29,30,31,32]. Srimathveeravalli et al. developed a robotic system that controls catheters with a mobile device and actuates the guidewire with friction wheels [33]. This drive mode may cause the guidewire to slip so that the slave robot cannot accurately replicate the movement of the master robot. Bao et al. developed a robotic system that adopts wire rope and gear as a linear and rotary driving device of guidewires and catheters [27]. However, the cable ropes are prone to generate stress and break at the bend and the gear transmission mechanism may affect the movement accuracy and cause pollution.

In order to obtain high motion accuracy, fast response, great cooperation performance, and MRI environment compatibility, piezo ultrasonic motors [34,35,36,37] can be implemented in RVIR systems. This paper proposes a novel RVIR based on a HUM and linear ultrasonic motor (LUM) which are not subject to electromagnetic interference and have the characteristic of rapid responsibility and high positional accuracy. In addition, using a HUM to drive the guidewire (0.014 inch, Nickel Titanium Alloy) and catheter (5 Fr, PVC) directly can eliminate the long transmission mechanism and ensure the catheter and guidewire have a good coaxial performance during surgery. As the core part of RVIR, a HUM was designed in detail and optimized. The structure of the slave robot and master robot are discussed in Section 2. The design and optimization of the HUM are analyzed experimentally and theoretically in Section 3. The key performances of RVIR, such as motion accuracy, master-slave tracking ability, and cooperative operation reliability, were tested and the results evaluated in Section 4.

## 2. System Design

### 2.1. Working Principle of RVIR

**Figure 1:**
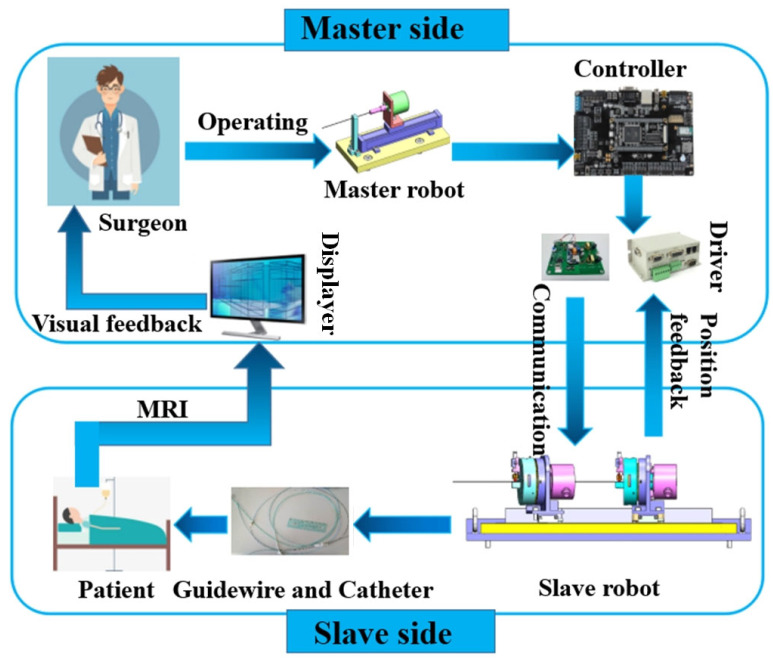
The schematic diagram of RVIR.

Figure 1 illustrates the working principle of the proposed RVIR. The master robot and slave robot are placed outside and inside the operating room, respectively, during surgery. On the master side, the rotary motion and linear motion of the guidewire manipulated by the surgeon are acquired and transmitted to the control unit (STM32F429IGT6, STMicro Co., Geneva, Switzerland) which drives the slave robot to control the guidewire and catheter to move in the blood vessel. The positional information of the guidewire and catheter is detected by MRI and the surgeon uses the imaging as visual feedback to proceed with the next motion.

### 2.2. Slave Robot Design

**Figure 2:**
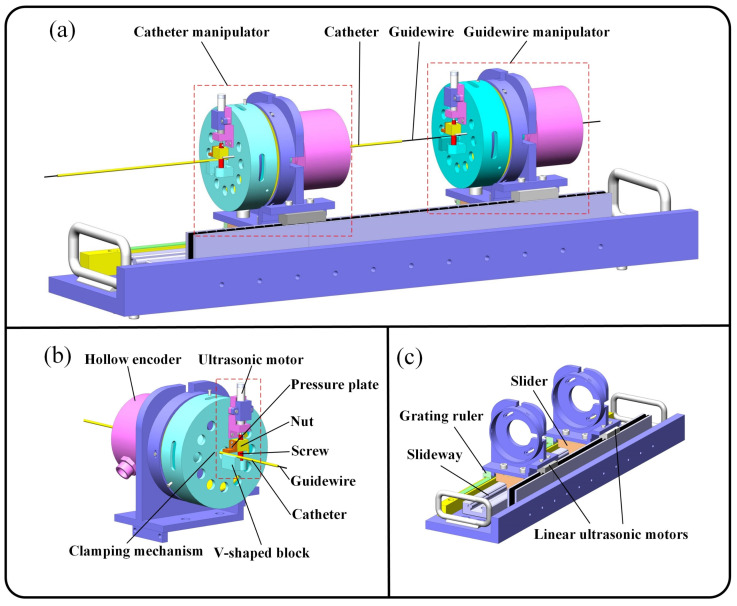
Main components of the slave robot (**a**), the structure of hollow mechanism (**b**), and linear motion platform (**c**).

As shown in Figure 2a, the slave robot mainly includes the GM and CM. The two manipulators are composed of a hollow mechanism and linear motion platform, shown in Figure 2b,c, that can drive the guidewire and catheter to rotate and deliver. In order to transfer the rotary motion of the HUM to the guidewire and catheter and realize precise positioning, a screw clamping mechanism was designed. The clamping mechanism uses a V-shaped block to position the guidewire and catheter and utilizes an ultrasonic motor to drive the screw nut to implement clamping. The linear motion platform can realize the linear motion of the guidewire and catheter by using the LUM to drive two hollow mechanisms. The rotation angle and linear displacement of guidewire and catheter were detected by a hollow encoder (HKT5612-301G-2000BZ1-5L, Rep-Avago Electronic Technology Co., Ltd., Wuxi, China) and grating ruler (SX-520-5-A, Fagor Automation Co., Ltd., Beijing, China), respectively, which can be used in MRI environment.

The hollow mechanism and LUM can directly transfer rotation motion and linear motion to the guidewire and catheter, avoiding the complex transmission structure and long-distance transmission channel, which can significantly improve the accuracy and response speed of master-slave tracking motion. At the same time, the direct-driven method and the positioning of the clamping mechanism can make the guidewire and catheter have good coaxiality during operation, which is conducive to cooperative control.

### 2.3. Master Robot Design

**Figure 3:**
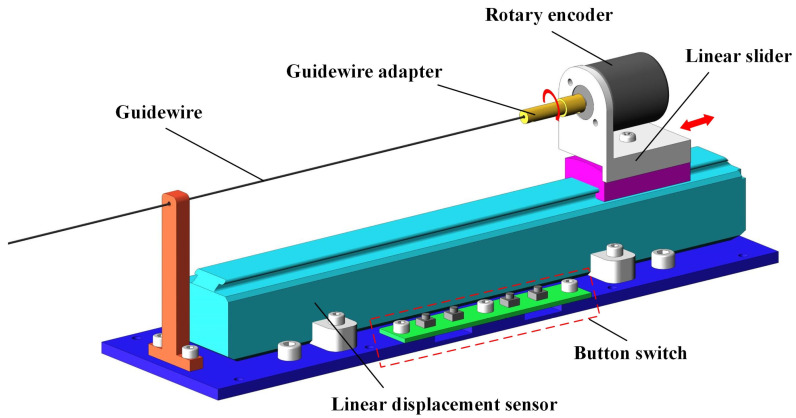
Main components of the master robot.

As shown in Figure 3, the master robot is a platform for the surgeon to operate during surgery. The linear motion and rotary motion of the master robot can be transferred to the slave robot through sensors and controllers to complete the corresponding operation. Considering the operation habit of the surgeon, a guidewire was introduced into the master robot as the control object.

The surgeon realizes the rotary motion and linear delivery motion of the guidewire in the master robot by rotating the guidewire adapter and moving the linear slider. The incremental rotary encoder (ZSP5208-001G-5000BZ1-5F, Ningbo fangjiaoshi Electrical Equipment Co., Ltd., Ningbo, China) and slider-type linear displacement sensor (1800110250A20, Germanjet Co., Ltd., Shenzhen, China) were used to record the rotary angle and displacement. The button switch was designed to select the guidewire or catheter in the slave robot as the control object.

## 3. Design and Optimization of the HUM

### 3.1. The Structure and Motion Mechanism of the HUM

**Figure 4:**
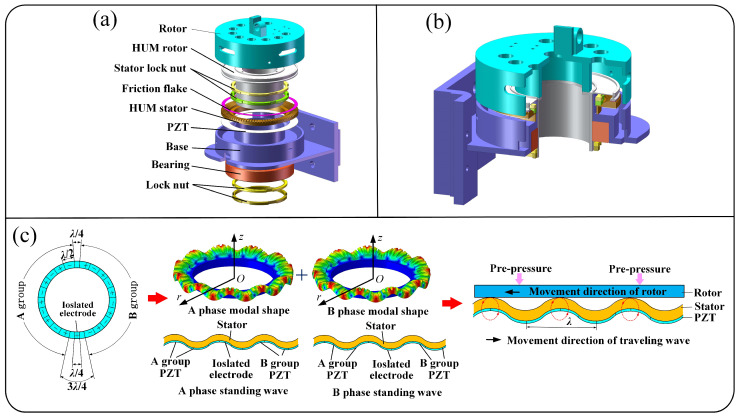
The explosion view (**a**) and section view (**b**) of the HUM. (**c**) The motion mechanism of the HUM.

Although there are many kinds of HUMs at present, if they are directly applied to RVIR, they will cause structural complexity and difficulty in meeting operating requirements. Therefore, we designed the HUM which is used in RVIR independently. The structure of the HUM is shown in Figure 4a,b. The HUM stator is fastened to the base by a stator lock nut and the stator, and the rotor is pressed together by a lock nut. As shown in Figure 4c, the HUM stator has two orthogonal modal shapes A and B and it can be expressed as:

(1) $$\left\{\begin{array}{l} \phi_{A}(r,\theta )=R(r)\sin(n\theta ),\\ \phi_{B}(r,\theta )=R(r)\cos(n\theta ), \end{array}\right.$$

where *R*(*r*) is the transverse displacement distribution function along the radial direction, sin(*nθ*) and cos(*nθ*) are the displacement distribution functions along the circumferential direction, and n is the number of nodal lines (*n* = 11 in this paper). When two-phase voltages are applied to the two-phase piezoelectric ceramic (PZT), respectively, and the interference modes are not considered, the two-phase A and B standing waves can be written as:

(2) $$\left\{\begin{array}{l} w_{A}(r,\theta ,t)=W_{A}R(r)\sin(n\theta )\cos(\omega_{n}t),\\ w_{B}(r,\theta ,t)=W_{B}R(r)\cos(n\theta )\cos(\omega_{n}t+\alpha ), \end{array}\right.$$

where *W*_A_ and *W*_B_ are the amplitudes of phase A and B standing waves, *α* is the phase difference between the two-phase responses. If the two-phase voltages are applied at the same time, according to the superposition principle, the displacement response of the stator is

(3) $$\begin{array}{l} w=w_{A}+w_{B}=[(W_{A}-W_{B}\sin\alpha )\sin(n\theta +\omega_{n}t)\\ \;\;\;\;\;\;\;\;\;\;\;\;\;\;\;\;\;\;\;\;\;\;\;\;+(W_{A}+W_{B}\sin\alpha )\sin(n\theta -\omega_{n}t)\\ \;\;\;\;\;\;\;\;\;\;\;\;\;\;\;\;\;\;\;\;\;\;\;\;+2W_{B}\cos\alpha \cos(n\theta )\cos(\omega_{n}t)]R(r)/2, \end{array}$$

when *α* = ±π/2 and *W*_A_ = *W*_B_ = *W*_0_, Equation (3) becomes the forward and backward traveling wave:

(4) $$\left\{\begin{array}{l} w(r,\theta ,t)=W_{0}R(r)\sin(n\theta -\omega_{n}t),\\ w(r,\theta ,t)=W_{0}R(r)\sin(n\theta +\omega_{n}t), \end{array}\right.$$

where *W*_0_ is the amplitude of the traveling wave. The traveling wave can make points on the stator from the ellipse motion trajectory which produces the rotation of the HUM rotor and then drives the guidewire and catheter, which are clamped, to rotate.

### 3.2. Optimization of the HUM

As the rotary driving mechanism of the guidewire and catheter, the mechanical properties of the HUM will significantly affect the control accuracy of RVIR. So, the adaptive genetic algorithm [38,39] which can obtain the approximate optimal solution in the whole domain was adopted to optimize the stator of the HUM for better performance. The optimization analysis process is shown in Table 1.

**Figure 5:**
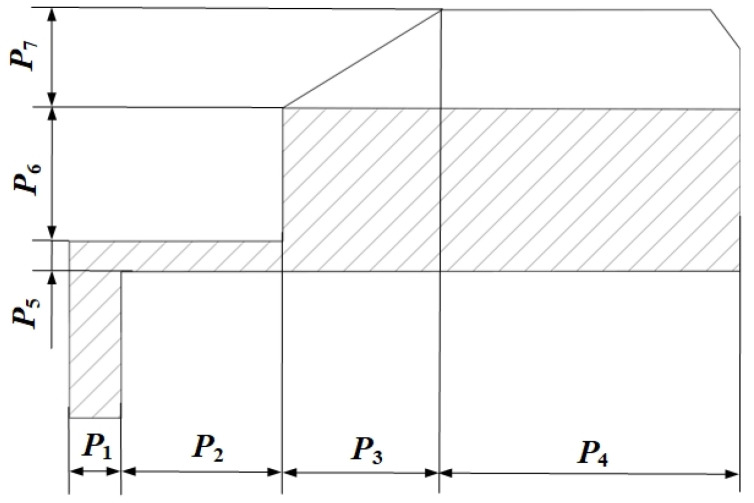
Parametric structure of the HUM stator.

Based on engineering experience and actual processing conditions, seven structure parameters of the HUM stator were selected as design variables, as shown in Figure 5, and the initial size and design space of each design variable was presented in Table 2.

The structure optimization of the HUM stator is mainly faced with four design requirements. The objective functions and constraint condition of optimization analysis can be obtained by describing each requirement mathematically:

(1) The two working mode frequencies, *F*_A_ and *F*_B_, should be as far away as possible from the frequencies of the two front and rear interference modes, *F*_f_ and *F*_b_. If the frequency of interference modes is close to that of working modes, the interference modes may be excited when two-phase voltages are applied. According to the superposition principle, the stator surface will produce an irregular motion pattern instead of an ideal traveling wave, which will reduce the stability and efficiency of motor operation, and thus reduce the motion accuracy of the guidewire and catheter. Therefore, the objective function *G*_1_ can be expressed as:

(5) $$G_{1}=\frac{1}{F_{A}-F_{f}}+\frac{1}{F_{b}-F_{B}}.$$

(2) Increasing the amplitude of the traveling wave can more easily push the rotor and improve the efficiency of the HUM, so the objective function *G*_2_ can be set as:

(6) $$G_{2}=\frac{1}{W_{0}}.$$

(3) The stress value of the stator inner ring should be as small as possible, otherwise, the vibration of the stator may be excessively limited by the fixed constraint, which will also reduce the motion precision of the HUM. The objective function *G*_3_ can be described as:

(7) $$G_{3}=\sigma_{in}.$$

(4) *σ*_res_ and *σ*_vib_ which are the maximum stress value of stator at rest and vibration should be less than yield stress *σ*_s_. The optimization constraint can be expressed as follows:

(8) $$max\left(\sigma_{res},\sigma_{vib}\right)<\sigma_{s}.$$

The multi-objective optimization problem can be converted into a single-objective optimization problem by combining multiple optimization objective functions into a total objective function *G* with linear weighting method, the expression can be written as:

(9) $$G(P)=\sum_{i=1}^{3}\beta_{i}G_{i},$$

where ***P*** = [*P*_1_,*P*_2_,*P*_3_,*P*_4_,*P*_5_,*P*_6_,*P*_7_]^T^ is the design variable vector, *β_i_* are the weight coefficients of each sub-objective function. In order to make the terms of objective function *G* dimensionless and have similar weight, the weight coefficients are set as:

(10) $$\beta_{1}=1\times 10^{2}Hz,\beta_{2}=3.3\times 10^{-7}m,\beta_{3}=1\times 10^{-8}Pa^{-1}.$$

According to the above analysis, the stator optimization model can be described as follows:

(11) $$\left\{\begin{array}{l} \underset{P}{min}G(P),\\ s.t.\left\{\begin{array}{l} P_{i}\in [P_{i}^{lb},P_{i}^{ub}]\\ max\left(\sigma_{sta},\sigma_{vib}\right)<\sigma_{s} \end{array}\right.,i=1,2,\ldots ,7, \end{array}\right.$$

where *P_i_^lb^* and *P_i_^ub^* are the lower and upper bounds of each design variable.

In order to improve the construction accuracy of response surface and sampling efficiency, the Latin hypercube random sampling method, which can make the sample points distribute evenly and cover the whole design space, was adopted. The sampling method is described in detail as follows:

In this paper, there are *M* = 7 design variables in total, and each design variable is assumed to follow a normal distribution. The mathematical expectation and standard deviations are the initial sizes and 1, respectively. The cumulative distribution function is *F_i_*(*P_i_*) and the total sampling times were *N* = 40. Firstly, the sampling situation of design variable *P_i_* is considered, and the interval [0,1] is divided into *N* sub-intervals. Random sampling is carried out in each sub-interval, and the original sampling value in the *k*_th_ interval can be obtained as follows:

(12) $$u_{k}=\frac{u}{N}+\frac{k-1}{N}.$$

where random variable *u* is uniformly distributed on [0,1], and the final sample value *P_ik_* corresponding to design variable *P_i_* is:

(13) $$P_{ik}=F_{i}^{-1}(u_{k})=F_{i}^{-1}(\frac{u+k-1}{N}).$$

Random sorting of *P_ik_* collected for *N* times can obtain the random sampling vector ***P**′_i_* = [*P′_i_*_1_, *P′_i_*_2_, …, *P^′^_iN_*]^T^ which represents the *N* sampling values of design variable *P_i_*. The random sampling vector of other design variables can be obtained in the same way.

Based on the above analysis, enough reasonable sample points were brought into the finite element model for analysis to be obtained. The response surface model was established after unreasonable sample points were filtered out according to the optimization constraint. The relationship between optimization objective function *G* and design variable vector ***P*** can be expressed as:

(14) $$G(P)=f(P)+\varepsilon =\sum_{i=0}^{m}\alpha_{i}\phi_{i}(P)+\varepsilon ,$$

where *f*(***P***) is the target approximation function used to describe response surface, *ε* and *m* are the statistical error and the number of basis functions, respectively, and *α_i_* and *ϕ_i_*(***P***) are the fitting coefficients and basis functions.

Considering the nonlinearity and analysis efficiency of the problem comprehensively, *f*(***P***) is described by a third-order polynomial:

(15) $$\begin{array}{l} f(P)=\gamma_{0}+\sum_{i=1}^{M}\gamma_{i}P_{i}+\sum_{i=1}^{M-1}\sum_{j=i+1}^{M}\gamma_{i,j}P_{i}P_{j}+\sum_{i=1}^{M}\gamma_{ii}P_{i}{}^{2}+\sum_{i=1}^{M-2}\sum_{j=i+1}^{M-1}\sum_{k=j+1}^{M}\gamma_{i,j,k}P_{i}P_{j}P_{k}\\ \;\;\;\;\;\;\;\;\;\;+\sum_{i=1}^{M}\sum_{j=1,j\neq i}^{M}\gamma_{ii,j}P_{i}{}^{2}P_{j}+\sum_{i=1}^{M}\gamma_{iii}P_{i}{}^{3}. \end{array}$$

The following equation can be obtained according to the least square principle:

(16) $$\left\{\begin{array}{l} Q(\alpha_{0},\alpha_{1},\ldots ,\alpha_{m})=\sum_{j=1}^{N}\varepsilon_{j}^{2}=\sum_{j=1}^{N}\left\{{\left[G(P_{j})-\sum_{i=0}^{m}\alpha_{i}\phi_{i}(P_{j})\right]}^{2}\right\},\\ \frac{\partial Q(\alpha_{0},\alpha_{1},\ldots ,\alpha_{m})}{\partial \alpha_{i}}=-2\Phi^{T}G+2\Phi^{T}\Phi \alpha =0,i=1,2,\ldots m, \end{array}\right.$$

where ***P****_j_* and *ε_j_* are the design variable vector and statistical error of the *j*_th_ sampling. **α** = [*α*_1_,*α*_2_,…,*α_m_*]^T^ is the undetermined regression coefficient vector. ***Φ*** and ***G*** are the basis function matrix and real response vector which can be expressed as:

(17) $$\left\{\begin{array}{l} \Phi =\left[\begin{array}{ccc} \phi_{1}(P_{1}) & \ldots & \phi_{m}(P_{1})\\ \vdots & & \vdots \\ \phi_{1}(P_{N}) & \ldots & \phi_{m}(P_{N}) \end{array}\right],\\ G={\left[f(P_{1}),f(P_{2}),\ldots ,f(P_{N})\right]}^{T}. \end{array}\right.$$

**Figure 6:**
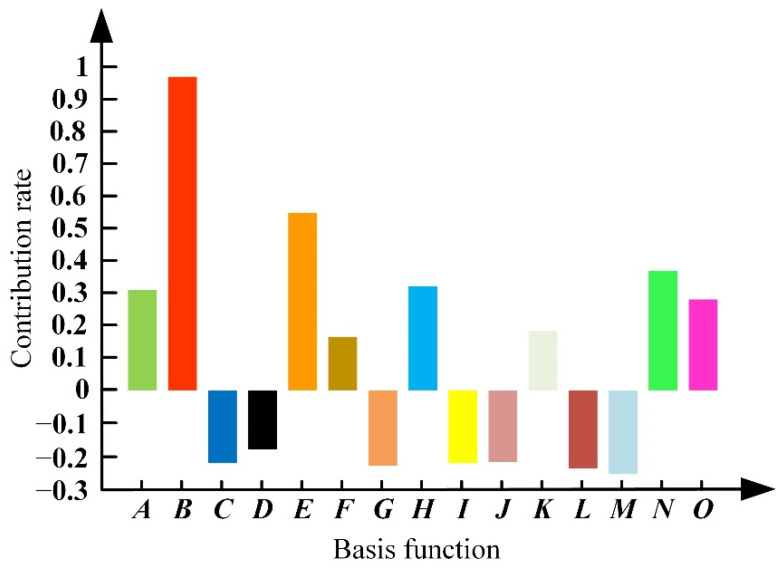
The contribution rate of basis functions.

According to Equations (16) and (17), **α** = (***Φ***^T^***Φ***)^−1^***Φ***^T^***G*** can be solved and the response surface model can be further obtained. However, *f*(***P***) includes a large number of design variables, high order terms, and cross terms, and its complexity makes analysis inefficient. Therefore, we get the contribution rate of each basis function to the target approximation function through sensitivity analysis, and use the objective function with a large contribution rate to construct the final response surface:

(18) $$\begin{array}{l} f(P)=0.990+0.275A+0.928B-0.224C\\ \;\;\;\;\;\;\;\;\;-0.147D+0.540E+0.157F-0.227G\\ \;\;\;\;\;\;\;\;\;+0.290H-0.212I-0.211J+0.189K\\ \;\;\;\;\;\;\;\;\;-0.235L-0.267M+0.353N+0.308O, \end{array}$$

where *A* = *P*_2_, *B* = *P*_6_, *C* = *P*_7_, *D* = *P*_6_*P*_7_, *E* = *P*_6_^2^, *F* = *P*_1_*P*_4_*P*_6_, *G* = *P*_3_*P*_5_*P*_6_, *H* = *P*_4_*P*_5_*P*_6_, *I* = *P*_1_^2^*P*_4_, *J* = *P*_2_^2^*P*_6_, *K* = *P*_5_^2^*P*_1_, *L* = *P*_5_^2^*P*_6_, and *M* = *P*_6_^2^*P*_2_, *N* = *P*_6_^2^*P*_5_, *O* = *P*_6_^3^. Figure 6 is the contribution rate of the basis functions which are used to construct the final response surface model.

**Figure 7:**
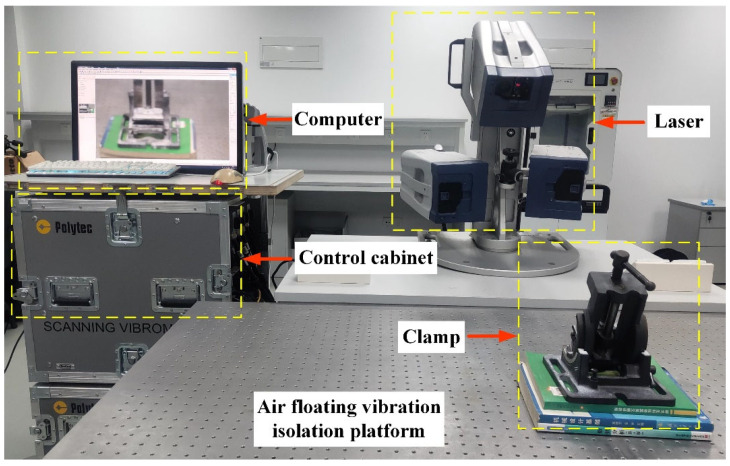
Vibration test platform for the optimized stator of the HUM.

The error determination coefficient is 94.5% which can illustrate the constructed response surface is able to describe the relationship between the optimization objective function *G* and the design variable vector ***P*** accurately. Through the adaptive genetic algorithm optimization analysis, we can obtain the optimized design variable vector ***P**_f_* is [0.83, 2.37, 2.4, 4.15, 0.55, 1.61, 1.35]^T^. The frequency differences between the working modes and the interference modes are 2.21 kHz and 1.91 kHz, which are much larger than the 0.43 kHz and 1.37 kHz under the initial design variable vector ***P**_i_*= [0.9, 2.5, 2.5, 4.5, 0.5, 1.5, 1.5]^T^. The interference modes of the optimized stator are far away from the working modes so the traveling wave can have better quality. The amplitude of the traveling wave is increased by nearly 3.5 times from 0.81 mm to 2.79 mm and the inner ring stress decreased from 15.23 Mpa to 11.17 Mpa. The result of FEA is verified by a vibration measurement experiment on the doppler 3D laser vibration measuring instrument as shown in Figure 7. The initial and optimized results are compared in Table 3.

## 4. Experimental Performance Evaluation

### 4.1. Experimental Platform and Control Algorithm

**Figure 8:**
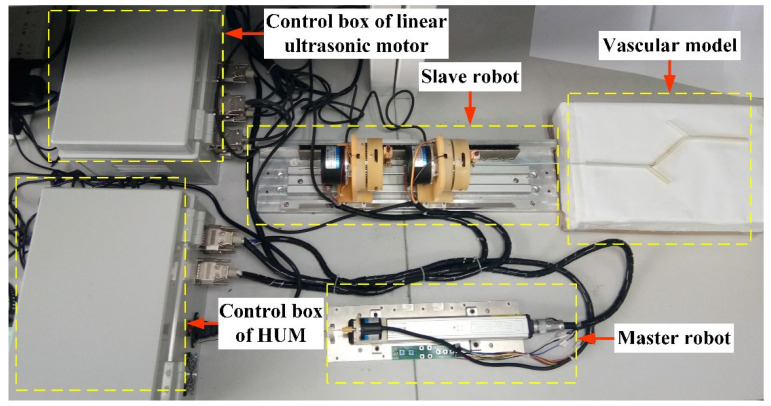
Experimental platform for testing RVIR performance.

In order to test the motion accuracy, verify master-slave tracking performance and cooperation performance, and simulate the vascular intervention process, four test experiments were conducted on the RVIR platform as shown in Figure 8. This section will describe the experimental methods and results of each test experiment in detail.

**Figure 9:**
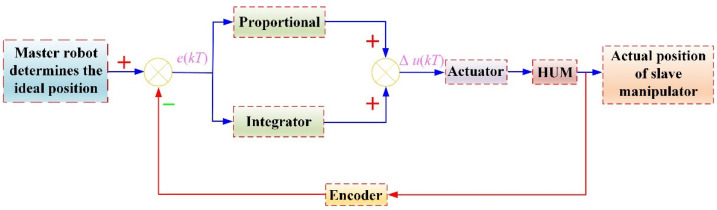
Control schematic of rotary motion.

Although the standard incremental PID control algorithm can eliminate the static error, the system will have a large overshoot at the initial stage of starting, so that the system may oscillate which will reduce the driving accuracy. As shown in Figure 9, the incremental integral separation PI algorithm was adopted to control the rotary motion of the guidewire and catheter in this paper. The master robot is used to determine the ideal position and the motor speed increment is calculated according to the position deviation through the PI algorithm. The driving frequency is determined by the frequency characteristic curve of the HUM, which is used as the input signal to adjust the rotation speed of the HUM through the actuator. When the position deviation is greater than the threshold value, the integrator will be canceled to avoid large overshooting and improve the response speed of the system. When the position deviation is small, the integrator is introduced to eliminate the static error and ensure the control accuracy of the system. The LUM adopted the same control algorithm to drive the GM and CM.

For rotary motion and linear motion, the threshold values are 5° and 2 mm, the proportional coefficient is 0.05 and 1.5, the integral coefficient is 0.05 and 1, respectively, and the sampling period is 20 ms.

### 4.2. Slave Robot Driving Accuracy Test Experiment

The motion of the HUM and LUM was directly controlled by the upper computer, instead of using a master-slave control algorithm, to verify the driving accuracy of the GM and CM in the slave robot. According to clinical experience, the GM and CM performed rotary motion and linear motion 20 times; each rotation angle and displacement distance were 30° and 25 mm and the speed of rotary motion and linear motion were 50°·s^−1^ and 20 mm·s^−1^, respectively. The motion errors of the GM and CM are shown in Table 4.

The experimental results show that the slave robot has good driving precision, the rotating motion accuracy, especially, of the GM and CM was very high and is far higher than the cable-driven mechanism [27], fully demonstrating the great advantage of applying the HUM to the structure design of RVIR in this study.

### 4.3. Master-Slave Tracking Test Experiment

**Figure 10:**
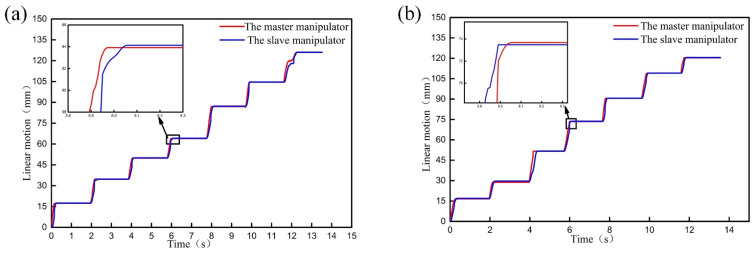

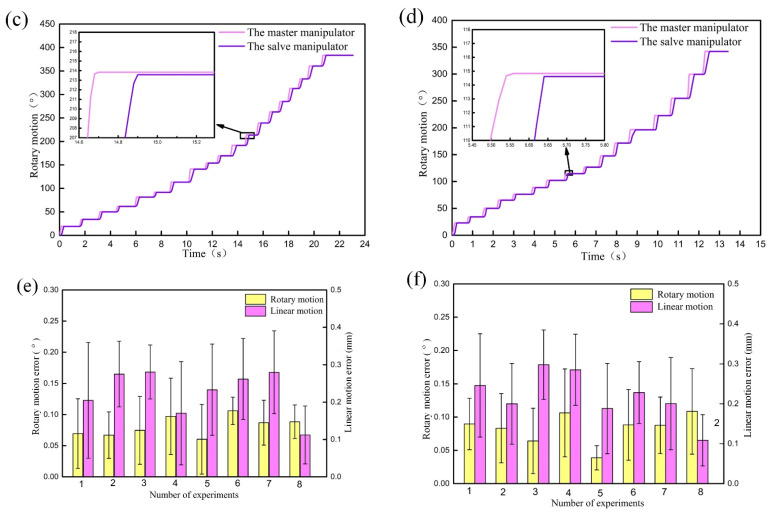
Linear tracking trajectory, rotary tracking trajectory, and tracking errors of the GM (**a**,**c**,**e**) and CM (**b**,**d**,**f**).

The master-slave tracking test was carried out to verify the control accuracy of the incremental integral separation PI control algorithm for RVIR. In order to verify the master-slave tracking ability of the GM and CM, eight linear motion and rotary motion tests were carried out, respectively. Taking the linear motion of the GM as an example, the linear motion of the GM was carried out by master-slave control mode many times. The master-slave tracking trajectories in the experimental process were recorded by sensors, and the motion errors of each experiment were calculated, as shown in Figure 10. The same method was used to test the master-slave performance of the rotary motion and the CM.

The result shows that the motion trajectories of the GM and CM in slave robots are basically consistent with the master robot. The tracking errors of the GM and CM are less than 0.3 mm and 0.15°, and the master-slave tracking lag time of the GM and CM is less than 200 ms. The results show that using incremental integral separation PI control algorithm to drive the HUM and LUM can make RVIR have good control accuracy and high response speed.

### 4.4. Cooperative Control Motion Test Experiment

**Figure 11:**
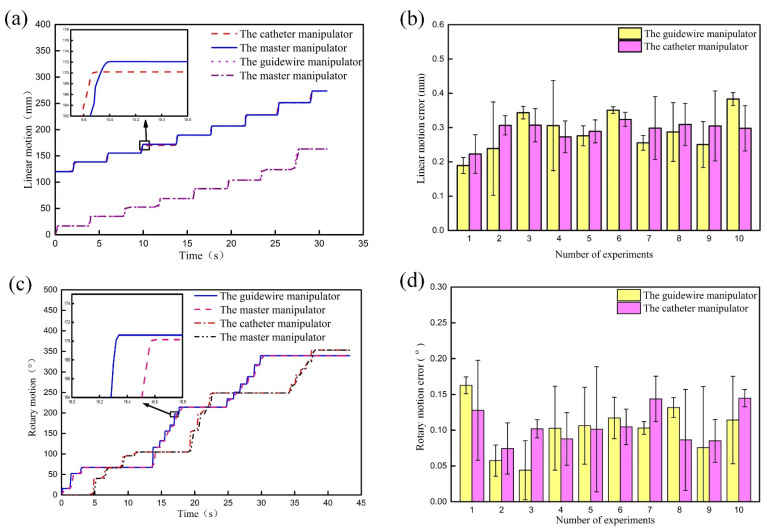
Tracking trajectories and tracking errors of linear motion (**a**,**b**) and rotary motion (**c**,**d**) during cooperation.

Complex vascular interventional surgery requires the cooperation of the guidewire and catheter, and the alternating linear motion and alternating rotary motion of guidewire and catheter are the main motion modes during the process of cooperative operation. So, this paper mainly studied the coupling degree of linear-linear motion and rotary-rotary motion between guidewire and catheter. Firstly, using the master-slave control mode to drive the GM to move a certain distance and then to drive the CM to move a certain distance. This process was repeated many times, and the linear motion tracking trajectories and linear motion errors of the GM and CM during the cooperation process was obtained as shown in Figure 11a,b. The test method of rotary motion is similar and the results are shown in Figure 11c,d.

Experimental results show that the linear motion and rotary motion errors are less than 0.4 mm and 0.2°, respectively, and the master-slave tracking lag time is less than 300 ms. Although the master-slave tracking errors and lag time during cooperating are greater than that in the master-slave tracking test experiment, the results are very close. The results indicate that there is no obvious motion coupling between the GM and CM, and RVIR can still maintain high motion accuracy during cooperation.

### 4.5. Vascular Intervention Simulation Experiment

**Figure 12:**
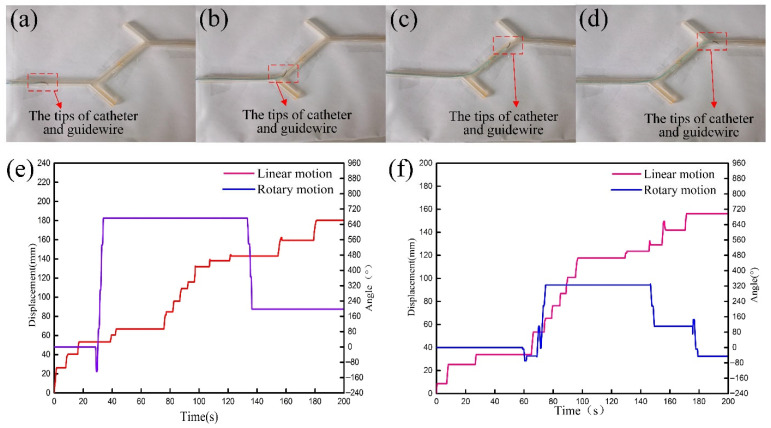
The movement of guidewire and catheter (**a**–**d**). The linear displacement and rotary angle of the GM (**e**) and CM (**f**).

In order to verify that RVIR can perform complex vascular interventional surgery, the simulation experiment was carried out. As shown in Figure 12a–d, the operator controlled the master robot to make the GM and CM drive the guidewire and catheter from their initial position, pass through two vessel branches, and finally reach the target position. Figure 12e,f shows the linear and rotary motion of the GM and CM. In this experiment, multiple experimenters were selected for operating to avoid contingency. The whole movement process of guidewire and catheter is clearly presented in the attached video.

The result shows that the experimenters were able to control the robot to successfully complete the vascular intervention simulation test, with the average operation time and maximum operation time of 161 s and 195 s, respectively, which demonstrates that RVIR has reliable and stable operation.

## 5. Conclusions and Future Work

In this paper, we proposed a novel RVIR that can complete complex vascular interventional surgery. A HUM and LUM were used to directly drive the guidewire and catheter which eliminated the redundant transmission mechanism in the traditional structure. At the same time, this RVIR enables the guidewire and catheter to maintain good coaxiality during surgery, which is beneficial to the cooperation. In addition, an adaptive genetic algorithm was used to optimize the stator of the HUM, so that the stator can produce more ideal traveling waves and significantly increase the amplitude of the traveling wave, which is conducive to improving the driving performance of the HUM. Finally, a slave robot driving accuracy test experiment, master-slave tracking test experiment, cooperative control motion test experiment, and vascular intervention simulation experiment were carried out to verify that the RVIR has good control accuracy, high master-slave tracking performance, and great cooperative control performance. In the future, we will conduct performance test experiments and vascular intervention simulation tests under an MRI environment to fully verify the great MRI compatibility of RVIR. Furthermore, the LUM will be optimized to further improve the linear motion accuracy of the GM and CM.

## Figures and Tables

**Table 1:** Optimization analysis process.

	Analysis Process
Step 1	Determine the optimal design space of the stator
Step 2	Establish the optimization model of the stator
Step 3	Use Latin hypercube sampling to obtain sample points
Step 4	Calculate the response of the sample points
Step 5	Filter data points
Step 6	Build response surface model
Step 7	Use a genetic algorithm to find the optimal solution in the whole domain

**Table 2:** Initial size and design space of the HUM stator.

Design Variable	Initial Size/mm	Design Space/mm
*P*_1_	0.9	0.81–0.99
*P*_2_	2.5	2.00–2.90
*P*_3_	2.5	2.25–2.75
*P*_4_	4.5	4.00–5.00
*P*_5_	0.5	0.42–0.58
*P*_6_	1.5	1.30–1.70
*P*_7_	1.5	1.35–1.65

**Table 3:** Comparison of experimental and FEA results.

		Frequency/kHz				Amplitude/μm	Stress/Mpa
		*F*_f_	*F*_b_	*F*_A_	*F*_B_	*W*_0_	*σ*_in_
FEA	Initial	37.25	39.05	38.62	38.62	0.81	15.23
	Optimized	36.52	40.64	38.43	38.43	2.79	11.17
TEST	Initial	37.11	39.01	38.55	38.55	0.93	—
	Optimized	36.37	40.53	38.43	38.43	3.12	—

**Table 4:** Slave robot driving accuracy test results.

Object	Motion	Mean	SD	Cumulation
GM	Linear/mm	0.12	0.035	0.57
	Rotary/°	0.08	0.027	0.35
CM	Linear/mm	0.13	0.029	0.45
	Rotary/°	0.11	0.023	0.27
